# 1-(Adamantan-1-ylcarbon­yl)-3-(2,6-difluoro-4-hy­droxy­phen­yl)thio­urea

**DOI:** 10.1107/S1600536812018806

**Published:** 2012-05-02

**Authors:** Shaaban K. Mohamed, Abdel-Aal M. Jaber, Sohail Saeed, Khuram Shahzad Ahmad, Wing-Tak Wong

**Affiliations:** a Chemistry and Environmental Division, Manchester Metropolitan University, Manchester M1 5GD, England; b Chemistry Department, Faculty of Science, Assiut University, Assiut, Egypt; c Department of Chemistry, Research Complex, Allama Iqbal Open University, Islamabad 44000, Pakistan; d Department of Chemistry, The University of Hong Kong, Pokfulam Road, Pokfulam, Hong Kong SAR, People’s Republic of China

## Abstract

In the title mol­ecule, C_18_H_20_F_2_N_2_O_2_S, the 2,6-difluoro-4-hy­droxy­phenyl ring and the carbonyl­thio­urea group are each essentially planar, with maximum deviations of atoms from their mean planes of 0.0113 (14) and 0.1017 (15) Å, respectively; the dihedral angle between these two planes is 71.03 (6)°. An intra­molecular N—H⋯O hydrogen bond occurs. In the crystal, N—H⋯O and O—H⋯S hydrogen bonds connect the mol­ecules into chains running diagonally across the *bc* plane. C—H⋯S and C—H⋯F contacts are also observed.

## Related literature

For background studies of thio­urea derivatives, see: Saeed *et al.* (2011 ▶). For a related structure, see: Saeed *et al.* (2010 ▶).
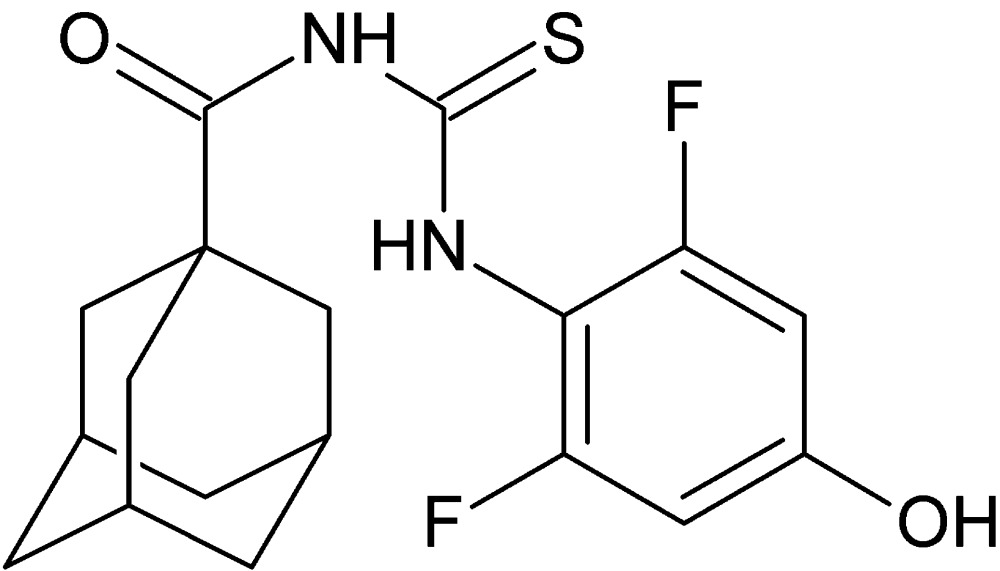


## Experimental

### 

#### Crystal data

C_18_H_20_F_2_N_2_O_2_S
*M* *_r_* = 366.42
Triclinic, 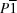

*a* = 7.3985 (9) Å
*b* = 10.4953 (13) Å
*c* = 12.4094 (15) Å
α = 65.554 (2)°
β = 79.372 (2)°
γ = 89.766 (2)°
*V* = 859.34 (18) Å^3^
*Z* = 2
Mo *K*α radiation
μ = 0.22 mm^−1^
*T* = 296 K
0.38 × 0.36 × 0.08 mm

#### Data collection

Bruker SMART 1000 CCD diffractometer
Absorption correction: multi-scan (*SADABS*; Sheldrick, 2004 ▶) *T*_min_ = 0.920, *T*_max_ = 0.982
4824 measured reflections
2976 independent reflections
2399 reflections with *I* > 2σ(*I*)
*R*_int_ = 0.010

#### Refinement

*R*[*F* ^2^ > 2σ(*F* ^2^)] = 0.038
*wR*(*F* ^2^) = 0.100
*S* = 1.04
2976 reflections
238 parameters
H atoms treated by a mixture of independent and constrained refinement
Δρ_max_ = 0.17 e Å^−3^
Δρ_min_ = −0.18 e Å^−3^

### 

Data collection: *SMART* (Bruker, 1998 ▶); cell refinement: *SAINT* (Bruker, 2006 ▶); data reduction: *SAINT*; program(s) used to solve structure: *SHELXS97* (Sheldrick, 2008 ▶); program(s) used to refine structure: *SHELXL97* (Sheldrick, 2008 ▶); molecular graphics: *Mercury* (Macrae *et al.*, 2008 ▶); software used to prepare material for publication: *SHELXL97*.

## Supplementary Material

Crystal structure: contains datablock(s) global, I. DOI: 10.1107/S1600536812018806/pv2537sup1.cif

Structure factors: contains datablock(s) I. DOI: 10.1107/S1600536812018806/pv2537Isup2.hkl

Supplementary material file. DOI: 10.1107/S1600536812018806/pv2537Isup3.cml

Additional supplementary materials:  crystallographic information; 3D view; checkCIF report

## Figures and Tables

**Table 1:** Hydrogen-bond geometry (Å, °)

*D*—H⋯*A*	*D*—H	H⋯*A*	*D*⋯*A*	*D*—H⋯*A*
N1—H1⋯O2	0.79 (2)	2.09 (2)	2.692 (2)	133 (2)
N1—H1⋯O2^i^	0.79 (2)	2.52 (2)	3.185 (3)	142 (2)
O1—H1*O*⋯S1^ii^	0.87 (3)	2.36 (3)	3.212 (2)	169 (3)
C14—H14*A*⋯S1^iii^	0.97	2.84	3.761 (2)	159
C14—H14*B*⋯F1^iv^	0.97	2.45	3.354 (3)	155

Symmetry codes: (i) 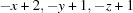; (ii) 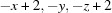; (iii) 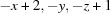; (iv) 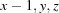.

**Fig. 1.**
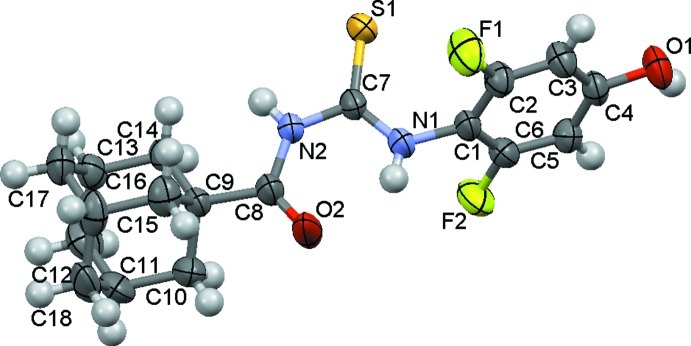
The molecular structure of the title compound with the atom numbering scheme. Displacement ellipsoids are drawn at the 50% probability level. H atoms are presented as small spheres of arbitrary radius.

**Fig. 2.**
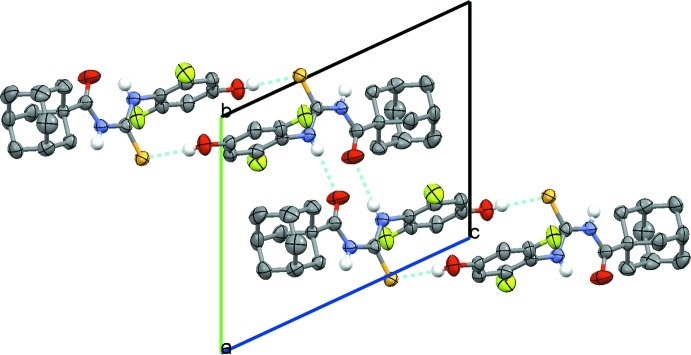
A view of the N—H···O and O—H···S hydrogen bonds (dotted lines) in the crystal structure of the title compound. H atoms non-participating in hydrogen-bonding were omitted for clarity.

## supplementary crystallographic information

### Comment

In continuation of our studies on structural chemistry of N,*N*'-disubstituted thiourea (Saeed, *et al.*, 2011), the structure of the title compound is described in this article.

In the title molecule (Fig. 1), the 2,6-difluoro-4-hydroxy-phenyl ring (C1–C6/O1/F1/F2) and the carbonyl thiourea group (S1/N1/N2/O2/C7/C8) are individually more or less planar, with maximum deviations of atoms from their mean planes being 0.0113 (14) Å for O1 and 0.1017 (15) Å for N2, respectively; the dihedral angle between these two planes is 71.03 (6)°.

Hydrogen bonding interactions were observed in the crystal lattice which connect the molecules into 1-D chains running diagonally across the *b-c*-plane (Table 1 and Fig. 2).

### Experimental

A mixture of adamantane-1-carbonyl chloride (199 mg, 1 mmol), 4-amino-3,5-difluorophenol (145 mg, 1 mmol) and potassium thiocyanate (100 mg) was heated for 30 minutes in ethanol (5 ml) at 351 K. The reaction mixture was left overnight to cool down at room temperature to afford a solid product which was filtered off, washed and recrystallized from ethanol. Colorless crystals suitable for X-ray crystallographic studies were collected and dried (yield = 82%; m. p. = 455-456 K).

### Refinement

The C-bound H atoms were placed at geometrically idealized positions with C—H = 0.93, 0.97 and 0.98 Å for phenyl, methylene and methine H-atoms, respectively, and were refined using riding model with *U*_iso_(H) = 1.2*U*_eq_(C). The N and O-bound H atoms were located from a difference Fourier map and were refined isotropically.

### Crystal data

**Table:**

C_18_H_20_F_2_N_2_O_2_S	*Z* = 2
*M**_r_* = 366.42	*F*(000) = 384
Triclinic, *P*1	*D*_x_ = 1.416 Mg m^−^^3^
Hall symbol: -P 1	Mo *K*α radiation, λ = 0.71073 Å
*a* = 7.3985 (9) Å	Cell parameters from 4824 reflections
*b* = 10.4953 (13) Å	θ = 2.8–25.0°
*c* = 12.4094 (15) Å	µ = 0.22 mm^−^^1^
α = 65.554 (2)°	*T* = 296 K
β = 79.372 (2)°	Plate, colourless
γ = 89.766 (2)°	0.38 × 0.36 × 0.08 mm
*V* = 859.34 (18) Å^3^	

### Data collection

**Table:**

Bruker SMART 1000 CCD diffractometer	2976 independent reflections
Radiation source: fine-focus sealed tube	2399 reflections with *I* > 2σ(*I*)
Graphite monochromator	*R*_int_ = 0.010
ω & φ scans	θ_max_ = 25.0°, θ_min_ = 2.8°
Absorption correction: multi-scan (*SADABS*; Sheldrick, 2004)	*h* = −8→8
*T*_min_ = 0.920, *T*_max_ = 0.982	*k* = −9→12
4824 measured reflections	*l* = −14→14

### Refinement

**Table:**

Refinement on *F*^2^	Primary atom site location: structure-invariant direct methods
Least-squares matrix: full	Secondary atom site location: difference Fourier map
*R*[*F*^2^ > 2σ(*F*^2^)] = 0.038	Hydrogen site location: inferred from neighbouring sites
*wR*(*F*^2^) = 0.100	H atoms treated by a mixture of independent and constrained refinement
*S* = 1.04	*w* = 1/[σ^2^(*F*_o_^2^) + (0.0409*P*)^2^ + 0.3498*P*] where *P* = (*F*_o_^2^ + 2*F*_c_^2^)/3
2976 reflections	(Δ/σ)_max_ < 0.001
238 parameters	Δρ_max_ = 0.17 e Å^−^^3^
0 restraints	Δρ_min_ = −0.18 e Å^−^^3^

### Special details

**Table:**

Geometry. All e.s.d.'s (except the e.s.d. in the dihedral angle between two l.s. planes) are estimated using the full covariance matrix. The cell e.s.d.'s are taken into account individually in the estimation of e.s.d.'s in distances, angles and torsion angles; correlations between e.s.d.'s in cell parameters are only used when they are defined by crystal symmetry. An approximate (isotropic) treatment of cell e.s.d.'s is used for estimating e.s.d.'s involving l.s. planes.
Refinement. Refinement of *F*^2^ against ALL reflections. The weighted *R*-factor *wR* and goodness of fit *S* are based on *F*^2^, conventional *R*-factors *R* are based on *F*, with *F* set to zero for negative *F*^2^. The threshold expression of *F*^2^ > σ(*F*^2^) is used only for calculating *R*-factors(gt) *etc*. and is not relevant to the choice of reflections for refinement. *R*-factors based on *F*^2^ are statistically about twice as large as those based on *F*, and *R*- factors based on ALL data will be even larger.

### Fractional atomic coordinates and isotropic or equivalent isotropic displacement parameters (Å^2^)

**Table:**

	*x*	*y*	*z*	*U*_iso_*/*U*_eq_	
S1	0.98449 (8)	−0.02031 (6)	0.68281 (5)	0.05290 (18)	
F1	1.3675 (2)	0.16253 (18)	0.66290 (12)	0.0801 (5)	
F2	0.81301 (19)	0.25665 (16)	0.85556 (13)	0.0769 (4)	
O1	1.2972 (3)	0.08462 (19)	1.06720 (15)	0.0659 (5)	
H1O	1.212 (4)	0.076 (3)	1.128 (3)	0.095 (11)*	
O2	0.8566 (3)	0.42734 (17)	0.47025 (18)	0.0918 (7)	
N1	1.0107 (3)	0.2470 (2)	0.64901 (16)	0.0533 (5)	
H1	0.993 (3)	0.327 (3)	0.613 (2)	0.061 (7)*	
N2	0.8692 (3)	0.19619 (19)	0.51851 (15)	0.0481 (4)	
H2	0.838 (3)	0.132 (2)	0.5017 (18)	0.046 (6)*	
C1	1.0863 (3)	0.2106 (2)	0.75458 (17)	0.0465 (5)	
C2	1.2611 (3)	0.1657 (2)	0.76211 (18)	0.0508 (5)	
C3	1.3321 (3)	0.1234 (2)	0.86477 (18)	0.0521 (5)	
H3	1.4501	0.0926	0.8662	0.063*	
C4	1.2233 (3)	0.1278 (2)	0.96622 (17)	0.0467 (5)	
C5	1.0489 (3)	0.1751 (2)	0.96344 (18)	0.0491 (5)	
H5	0.9768	0.1803	1.0311	0.059*	
C6	0.9851 (3)	0.2141 (2)	0.85798 (19)	0.0484 (5)	
C7	0.9559 (3)	0.1514 (2)	0.61553 (16)	0.0433 (5)	
C8	0.8121 (3)	0.3264 (2)	0.45461 (19)	0.0517 (5)	
C9	0.6926 (3)	0.3338 (2)	0.36480 (16)	0.0417 (4)	
C10	0.5468 (3)	0.4380 (2)	0.3671 (2)	0.0615 (6)	
H10A	0.4691	0.4041	0.4471	0.074*	
H10B	0.6072	0.5285	0.3488	0.074*	
C11	0.4287 (4)	0.4539 (3)	0.2740 (3)	0.0733 (8)	
H11	0.3357	0.5200	0.2761	0.088*	
C12	0.3332 (3)	0.3132 (3)	0.3014 (3)	0.0759 (8)	
H12A	0.2537	0.2768	0.3810	0.091*	
H12B	0.2573	0.3246	0.2426	0.091*	
C13	0.4766 (3)	0.2112 (2)	0.2971 (2)	0.0541 (5)	
H13	0.4143	0.1204	0.3151	0.065*	
C14	0.5933 (3)	0.1922 (2)	0.39204 (18)	0.0457 (5)	
H14A	0.6834	0.1251	0.3912	0.055*	
H14B	0.5145	0.1559	0.4718	0.055*	
C15	0.8154 (3)	0.3888 (3)	0.23861 (19)	0.0575 (6)	
H15A	0.9072	0.3232	0.2363	0.069*	
H15B	0.8793	0.4783	0.2197	0.069*	
C16	0.6960 (4)	0.4067 (3)	0.1452 (2)	0.0684 (7)	
H16	0.7744	0.4432	0.0643	0.082*	
C17	0.6014 (3)	0.2650 (3)	0.1738 (2)	0.0631 (6)	
H17A	0.5297	0.2744	0.1133	0.076*	
H17B	0.6932	0.1990	0.1728	0.076*	
C18	0.5517 (4)	0.5102 (3)	0.1499 (2)	0.0811 (9)	
H18A	0.6129	0.6003	0.1320	0.097*	
H18B	0.4778	0.5238	0.0896	0.097*	

### Atomic displacement parameters (Å^2^)

**Table:**

	*U*^11^	*U*^22^	*U*^33^	*U*^12^	*U*^13^	*U*^23^
S1	0.0726 (4)	0.0439 (3)	0.0474 (3)	0.0169 (3)	−0.0265 (3)	−0.0185 (2)
F1	0.0762 (10)	0.1176 (13)	0.0479 (8)	0.0208 (9)	−0.0104 (7)	−0.0372 (8)
F2	0.0675 (9)	0.0973 (11)	0.0828 (10)	0.0420 (8)	−0.0378 (8)	−0.0454 (9)
O1	0.0662 (11)	0.0889 (13)	0.0456 (9)	0.0123 (9)	−0.0283 (8)	−0.0240 (9)
O2	0.1500 (18)	0.0459 (10)	0.1119 (15)	0.0241 (10)	−0.0993 (14)	−0.0348 (10)
N1	0.0806 (14)	0.0408 (10)	0.0478 (10)	0.0158 (10)	−0.0380 (10)	−0.0175 (9)
N2	0.0674 (12)	0.0421 (10)	0.0468 (10)	0.0137 (9)	−0.0317 (9)	−0.0221 (8)
C1	0.0626 (13)	0.0406 (11)	0.0417 (11)	0.0084 (10)	−0.0255 (10)	−0.0165 (9)
C2	0.0556 (13)	0.0596 (13)	0.0379 (11)	0.0067 (10)	−0.0114 (9)	−0.0203 (10)
C3	0.0463 (12)	0.0630 (14)	0.0465 (12)	0.0087 (10)	−0.0163 (9)	−0.0194 (10)
C4	0.0523 (12)	0.0483 (12)	0.0398 (11)	0.0010 (9)	−0.0199 (9)	−0.0142 (9)
C5	0.0561 (13)	0.0529 (12)	0.0414 (11)	0.0070 (10)	−0.0134 (9)	−0.0214 (10)
C6	0.0515 (12)	0.0458 (11)	0.0549 (12)	0.0139 (9)	−0.0240 (10)	−0.0226 (10)
C7	0.0497 (11)	0.0473 (11)	0.0370 (10)	0.0114 (9)	−0.0172 (9)	−0.0183 (9)
C8	0.0689 (14)	0.0439 (12)	0.0497 (12)	0.0104 (10)	−0.0311 (11)	−0.0192 (10)
C9	0.0494 (11)	0.0394 (10)	0.0394 (10)	0.0077 (9)	−0.0197 (9)	−0.0152 (9)
C10	0.0797 (16)	0.0561 (14)	0.0650 (14)	0.0285 (12)	−0.0339 (12)	−0.0335 (12)
C11	0.0812 (18)	0.0689 (17)	0.0902 (19)	0.0416 (14)	−0.0524 (16)	−0.0393 (15)
C12	0.0513 (14)	0.090 (2)	0.096 (2)	0.0159 (13)	−0.0330 (14)	−0.0404 (16)
C13	0.0497 (12)	0.0524 (13)	0.0647 (14)	0.0012 (10)	−0.0258 (11)	−0.0232 (11)
C14	0.0472 (11)	0.0448 (11)	0.0428 (11)	0.0029 (9)	−0.0128 (9)	−0.0145 (9)
C15	0.0553 (13)	0.0594 (14)	0.0500 (12)	−0.0110 (11)	−0.0106 (10)	−0.0152 (11)
C16	0.0849 (18)	0.0748 (17)	0.0357 (11)	−0.0125 (14)	−0.0149 (11)	−0.0124 (11)
C17	0.0784 (16)	0.0709 (16)	0.0530 (13)	0.0091 (13)	−0.0310 (12)	−0.0314 (12)
C18	0.124 (2)	0.0520 (14)	0.0721 (17)	0.0101 (15)	−0.0662 (17)	−0.0112 (13)

### Geometric parameters (Å, º)

**Table:**

S1—C7	1.675 (2)	C10—C11	1.529 (3)
F1—C2	1.346 (2)	C10—H10A	0.9700
F2—C6	1.349 (2)	C10—H10B	0.9700
O1—C4	1.362 (2)	C11—C18	1.512 (4)
O1—H1O	0.87 (3)	C11—C12	1.517 (4)
O2—C8	1.211 (2)	C11—H11	0.9800
N1—C7	1.325 (3)	C12—C13	1.514 (3)
N1—C1	1.425 (2)	C12—H12A	0.9700
N1—H1	0.79 (2)	C12—H12B	0.9700
N2—C7	1.377 (2)	C13—C17	1.510 (3)
N2—C8	1.377 (3)	C13—C14	1.534 (3)
N2—H2	0.83 (2)	C13—H13	0.9800
C1—C6	1.376 (3)	C14—H14A	0.9700
C1—C2	1.380 (3)	C14—H14B	0.9700
C2—C3	1.369 (3)	C15—C16	1.535 (3)
C3—C4	1.382 (3)	C15—H15A	0.9700
C3—H3	0.9300	C15—H15B	0.9700
C4—C5	1.382 (3)	C16—C17	1.519 (3)
C5—C6	1.373 (3)	C16—C18	1.531 (4)
C5—H5	0.9300	C16—H16	0.9800
C8—C9	1.523 (2)	C17—H17A	0.9700
C9—C15	1.531 (3)	C17—H17B	0.9700
C9—C10	1.538 (3)	C18—H18A	0.9700
C9—C14	1.538 (3)	C18—H18B	0.9700
			
C4—O1—H1O	109 (2)	C18—C11—H11	109.4
C7—N1—C1	122.30 (17)	C12—C11—H11	109.4
C7—N1—H1	119.5 (17)	C10—C11—H11	109.4
C1—N1—H1	118.0 (17)	C13—C12—C11	109.5 (2)
C7—N2—C8	129.89 (18)	C13—C12—H12A	109.8
C7—N2—H2	113.5 (14)	C11—C12—H12A	109.8
C8—N2—H2	116.3 (14)	C13—C12—H12B	109.8
C6—C1—C2	115.57 (17)	C11—C12—H12B	109.8
C6—C1—N1	121.27 (19)	H12A—C12—H12B	108.2
C2—C1—N1	123.14 (19)	C17—C13—C12	110.9 (2)
F1—C2—C3	118.07 (19)	C17—C13—C14	109.09 (17)
F1—C2—C1	118.10 (17)	C12—C13—C14	109.23 (19)
C3—C2—C1	123.83 (19)	C17—C13—H13	109.2
C2—C3—C4	118.00 (19)	C12—C13—H13	109.2
C2—C3—H3	121.0	C14—C13—H13	109.2
C4—C3—H3	121.0	C13—C14—C9	109.91 (16)
O1—C4—C5	122.39 (19)	C13—C14—H14A	109.7
O1—C4—C3	116.74 (19)	C9—C14—H14A	109.7
C5—C4—C3	120.87 (17)	C13—C14—H14B	109.7
C6—C5—C4	118.13 (19)	C9—C14—H14B	109.7
C6—C5—H5	120.9	H14A—C14—H14B	108.2
C4—C5—H5	120.9	C9—C15—C16	109.42 (18)
F2—C6—C5	118.48 (19)	C9—C15—H15A	109.8
F2—C6—C1	117.93 (17)	C16—C15—H15A	109.8
C5—C6—C1	123.58 (19)	C9—C15—H15B	109.8
N1—C7—N2	117.75 (17)	C16—C15—H15B	109.8
N1—C7—S1	124.67 (14)	H15A—C15—H15B	108.2
N2—C7—S1	117.59 (15)	C17—C16—C18	110.1 (2)
O2—C8—N2	120.81 (18)	C17—C16—C15	109.17 (19)
O2—C8—C9	123.18 (18)	C18—C16—C15	109.3 (2)
N2—C8—C9	116.01 (17)	C17—C16—H16	109.4
C8—C9—C15	108.41 (17)	C18—C16—H16	109.4
C8—C9—C10	107.81 (16)	C15—C16—H16	109.4
C15—C9—C10	109.33 (17)	C13—C17—C16	109.20 (19)
C8—C9—C14	114.03 (15)	C13—C17—H17A	109.8
C15—C9—C14	108.57 (16)	C16—C17—H17A	109.8
C10—C9—C14	108.62 (17)	C13—C17—H17B	109.8
C11—C10—C9	109.65 (17)	C16—C17—H17B	109.8
C11—C10—H10A	109.7	H17A—C17—H17B	108.3
C9—C10—H10A	109.7	C11—C18—C16	109.77 (19)
C11—C10—H10B	109.7	C11—C18—H18A	109.7
C9—C10—H10B	109.7	C16—C18—H18A	109.7
H10A—C10—H10B	108.2	C11—C18—H18B	109.7
C18—C11—C12	109.5 (2)	C16—C18—H18B	109.7
C18—C11—C10	108.9 (2)	H18A—C18—H18B	108.2
C12—C11—C10	110.4 (2)		

### Hydrogen-bond geometry (Å, º)

**Table:**

*D*—H···*A*	*D*—H	H···*A*	*D*···*A*	*D*—H···*A*
N1—H1···O2	0.79 (2)	2.09 (2)	2.692 (2)	133 (2)
N1—H1···O2^i^	0.79 (2)	2.52 (2)	3.185 (3)	142 (2)
O1—H1*O*···S1^ii^	0.87 (3)	2.36 (3)	3.212 (2)	169 (3)
C14—H14*A*···S1^iii^	0.97	2.84	3.761 (2)	159
C14—H14*B*···F1^iv^	0.97	2.45	3.354 (3)	155

Symmetry codes: (i) −*x*+2, −*y*+1, −*z*+1; (ii) −*x*+2, −*y*, −*z*+2; (iii) −*x*+2, −*y*, −*z*+1; (iv) *x*−1, *y*, *z*.
